# Rehabilitation of Adult Patients with Primary Brain Tumors: A Narrative Review

**DOI:** 10.3390/brainsci10080492

**Published:** 2020-07-29

**Authors:** Parth Thakkar, Brian D. Greenwald, Palak Patel

**Affiliations:** 1 Rutgers Robert Wood Johnson Medical School, Piscataway, NJ 08854, USA; pt205@rwjms.rutgers.edu (P.T.); prp74@rwjms.rutgers.edu (P.P.); 2 JFK Johnson Rehabilitation Institute, Edison, NJ 08820, USA

**Keywords:** primary brain tumor, neuro-oncology, brain cancer, rehabilitation

## Abstract

Rehabilitative measures have been shown to benefit patients with primary brain tumors (PBT). To provide a high quality of care, clinicians should be aware of common challenges in this population including a variety of medical complications, symptoms, and impairments, such as headaches, seizures, cognitive deficits, fatigue, and mood changes. By taking communication and family training into consideration, clinicians can provide integrated and patient-centered care to this population. This article looks to review the current literature in outpatient and inpatient rehabilitation options for adult patients with PBTs as well as explore the role of the interdisciplinary team in providing survivorship care.

## 1. Introduction

About 80,000 people in the United States are diagnosed with a primary brain tumor (PBT) every year [1]. PBTs vary widely in their clinical presentations and can carry devastating prognoses for patients. The average survival rate for patients with malignant brain tumors is 35%, and the five-year survival rate for glioblastoma multiforme, the most common form of primary malignant brain tumor in adults, is 5.6%. There are an estimated 700,000 Americans living with a PBT, of which approximately 70% are benign and 30% are malignant [2]. Estimates for brain metastases vary widely due to a lack of evidence [3]. Incidence of individual tumor types can be seen in Table 1.

Although benign tumors are more prevalent and are considered more treatable than malignant tumors, intervention for any brain tumor can be invasive. PBTs can be treated in several ways, including surveillance, surgery, chemotherapy, and radiotherapy. The clinical approach should be considered based on a number of factors, including the qualities and location of the tumor along with the condition and preferences of the patients [4]. Treatments are not without risks, however. Radiotherapy can bring about fatigue and encephalopathy, and chemotherapy regimens can have many side effects as well [5,6,7,8,9,10]. Medical complications of patients with PBTs have been well documented and include venous thromboembolic disease, syndrome of inappropriate antidiuretic hormone (SIADH), dysphagia, and seizures, among others [11,12,13,14]. Psychiatric symptoms such as depression, fatigue, mood changes, and personality changes have been noted in conjunction with other symptoms such as headaches, sleep changes, and cognitive disturbances [15,16,17,18,19,20,21].

Survivorship care models propose interdisciplinary healthcare teams to deliver tailored care to cancer patients [22,23,24,25]. In this spectrum of care, rehabilitative measures have shown to be beneficial in both the inpatient and outpatient rehabilitation settings [26,27]. Medical complications cause barriers in both rehabilitation and broader healthcare settings. In rehabilitation, healthcare providers face the challenges of treating the primary neuromuscular pathology as well as the complex symptomology listed above. In order to achieve the functional goals of patients, care providers must understand how to manage these conditions. This review aims to guide clinicians treating adult patients with PBTs. Included are overviews of common complications and symptoms, survivorship care models and hospice care, family training, communication practices, and an examination of current literature on both outpatient and inpatient rehabilitation practices for this population.

## 2. Methods

**Figure 1:**
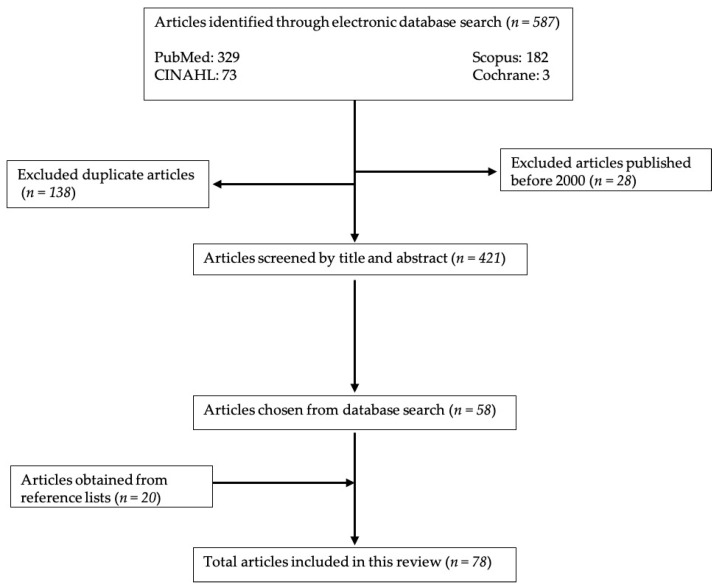
Methods process flow chart.

References for this narrative review were obtained by searching the online databases PUBMED, CINAHL, Cochrane, and Scopus. The search terms included: primary, brain, tumor, rehabilitation, with the Boolean operator “AND” to narrow results and connect terms. Articles published before the year 2000 were excluded from the search unless the research was important for this paper and not covered in more recent research. Only papers printed in or translated into English were included. Some of the references were not found through online databases but instead through reference lists of other articles. Please see Figure 1 for specifics about the article selection process. This narrative review does not aim to be a comprehensive review of the literature available on the topic of primary brain tumor rehabilitation. Instead, we focus on important articles that can guide providers in caring for PBT patients. Articles that discussed common medical complications and neuropsychiatric sequelae were included, as they outline barriers that patients face and must manage in rehabilitation settings.

## 3. Medical Complications of PBT Patients

### 3.1. Chemotherapy Side Effects

Temozolomide is an oral prodrug that can pass the blood–brain barrier and is rapidly converted to an alkylating metabolite that has cytotoxic effects. It has been shown to have a statistically significant survival benefit in glioblastoma patients when used with radiotherapy [6]. It also has an FDA indication for refractory anaplastic astrocytoma, along with off-label uses for other primary and metastatic malignancies. Common adverse reactions to temozolomide include myelotoxicity, nausea and vomiting, constipation, alopecia, fatigue, headaches, and seizures. Fatigue is the most commonly reported side effect [7]. It is notable to report that many of the symptoms of temozolomide overlap with symptoms of advancing primary brain tumors. In certain molecular subtypes of glioblastoma, lomustine is a chemotherapeutic agent that has been used in conjunction with temozolomide and radiotherapy. Lomustine crosses the blood–brain barrier and its mechanism of action is the alkylation and carbamylation of DNA and RNA [8]. Common adverse reactions include nausea, vomiting, leukopenia, bone marrow depression, and thrombocytopenia. Other treatments that are being considered in this population include bevacizumab and irinotecan [9].

Certain oligodendrogliomas have shown to be responsive to treatments of not only temozolomide but also a separate regimen of procarbazine, lomustine, and vincristine. In this three-drug regimen, common adverse reactions included diarrhea, liver and bone marrow toxicity, and fatigue [10].

### 3.2. Radiotherapy Side Effects

Therapeutic radiotherapy has been linked to several complications. Traditionally, these are categorized temporally in relation to the treatment [5,28]. Earlier complications are generally viewed as reversible while later complications are generally viewed as progressive and irreversible [28]. Acute encephalopathy occurs within several days and is characterized by symptoms such as nausea, somnolence, fever, headache, and exacerbation of focal symptoms. It typically responds to an increased dose of steroids [5].

Early delayed encephalopathy is a broad term to describe sequelae occurring weeks to months after treatment.

- One of these syndromes is “pseudoprogression”, a complication that occurs in 25% of glioblastoma patients receiving concomitant temozolomide. Within 2 months of radiotherapy, MRI imaging shows worsening enhancement, and some patients have worsening neurologic symptoms. Although the exact mechanism is not known, it is believed to be caused by an interaction between the temozolomide and radiotherapy.
- Symptomatic or asymptomatic increase in contrast enhancement on repeat imaging can occur within 3–9 months after the initiation of radiotherapy. Symptoms typically improve over the course of the months and can be further helped with the use of steroids.
- Multifocal MRI lesions are rare but can occur 6–36 months after therapy and are highly variable in course. They can be asymptomatic or present with worsening focal deficits and are characterized as either stabilizing, progressing, or spontaneously resolving.

Delayed encephalopathy can occur 6 months to a year after initial therapy and presents as focal necrosis. This necrosis can be life-threatening and sometimes responds with clinical and radiographic improvement to dexamethasone, but the effects of this treatment are believed only to be temporary and can lead patients to become steroid-dependent [5].

### 3.3. Corticosteroids

Glucocorticosteroids have been used for decades in the management of tumor-associated edema and symptomatology. They have been shown to be effective in reducing tumor-associated pain, nausea, and vomiting, and help improve appetite in cancer patients. In neuro-oncology, the steroid of choice is usually dexamethasone due to its long half-life, low mineralocorticoid action, and reduced chance of inducing psychosis. About 70% of patients with cerebral tumors report symptomatic improvement while using steroids. Steroids are also administered prior to elective surgeries to improve clinical outcomes by reducing postoperative edema. However, adverse effects include cushingoid habitus, easy bruising, immunosuppression, hypertension, glucose intolerance, electrolyte disturbances, gastrointestinal bleeding, osteoporosis, avascular necrosis, and more. The gastrointestinal complications, myopathy, Pneumocystis jiroveci pneumonitis (PJP) secondary to immunosuppression, and osteoporosis are of specific concern for patients with brain tumors. Since many of these side effects are dose-dependent, it is important to have patients on the lowest effective dose possible [29].

### 3.4. Seizures

Seizures are a known complication of brain tumors and can affect patients’ quality of life. They occur at variable rates depending on type and location of tumor and have been noted to be as high as 70–80% in glioneuronal tumors. After tumor resection, however, as many as 60–90% of patients no longer suffer from seizures [14]. In a study overviewing the long-term seizure outcomes in patients undergoing surgical resection for malignant astrocytomas, it was found that the majority of patients could achieve relief from seizures by a regimen of antiepileptic medications. Surgical resection offered most patients freedom from seizures in 6- and 12-month follow up periods. Factors that are positively associated with the occurrence of seizures are temporal lobe involvement and cortical location, and factors that are negatively associated with seizures are greater age, larger tumor size, and parietal lobe involvement [30]. In certain subgroups of patients with high-grade gliomas, seizures are an indicator of disease progression [31]. Prophylactic anticonvulsants are not recommended outside of the initial postoperative period [4].

### 3.5. Syndrome of Inappropriate Antidiuretic Hormone (SIADH)

SIADH has been observed in patients with primary brain tumors, and the resulting hyponatremia can manifest as headaches, vomiting, delirium, seizures, and coma. Edema of the brain could lead to fatal herniation [32]. In examining specifically for malignancy associated SIADH, short-term hyponatremia correction was associated with better survival, and a lower serum sodium concentration was associated with shorter median survival times. This study, however, did not specifically consider the population of patients with PBTs [12]. It is important to note that there is danger in overcorrecting sodium levels in hyponatremia which could lead to permanent and devastating brain damage [32]. Iatrogenic causes of SIADH should also be considered in the differential, specifically medications such as chlorpropamide, carbamazepine, oxcarbazepine, selective serotonin reuptake inhibitors, and intravenous cyclophosphamide.

### 3.6. Dysphagia

Difficulty swallowing, or dysphagia, can occur in a PBT patient either as a result of the lesion or as a complication of brain surgery and has been reported in 63% of PBT patients admitted to inpatient rehabilitation [13,33]. In addition to quality of life concerns, dysphagia also leads to medical complications of aspiration pneumonia, malnutrition, and dehydration. Incidence of these events can be decreased through rehabilitation specific to swallowing, as functional dysphagia improvements have been reported [13]. Patients with infratentorial tumors have dysphagia at higher frequencies than those with supratentorial lesions and no difference in frequency was noted between malignant and benign tumors [34].

### 3.7. Venous Thromboembolic Disease

Thromboembolic disease is a complication that can lead to serious detriment. Cancer patients have an increased risk of thromboembolism, and in malignant glioma patients it has been documented at a rate of 3–60% in the 6-week postoperative period [11]. Patients with cancer should receive prophylaxis as early as can safely be instituted after surgery, and it should continue for 7 to 10 days. The use of routine prophylaxis in an outpatient setting is not recommended, and novel oral anticoagulants have not been sufficiently studied in this population to warrant a recommendation [35].

## 4. Commonly Presenting Symptoms in PBT Populations

### 4.1. Headache

Patients with primary CNS tumors often experience headaches, although the type of headache may vary according to the location of the tumor. In a study of 111 patients with brain tumors, roughly 50% of patients experienced headaches. Of these patients, 77% had tension-type headaches and 68% expressed symptoms in the frontal area [36]. The pathophysiology of these headaches is believed to be secondary to the traction of structures such as arteries, veins, venous sinuses, cranial nerves, and portions of the dura mater [18]. Steroids have been used to curb edema caused by the tumor and subsequently offer relief, and surgery can also correct intracranial pressure. Post craniotomy headaches are typically characterized as pain around the surgical site and they typically respond to analgesics [37].

### 4.2. Cognitive Changes

Cognition refers to higher-order executive function and includes attention, memory, and reasoning. Patients with PBTs may experience deficits in cognition due to not only the presence and location of the tumor, but also as a side effect of treatments such as radiation, chemotherapy, and surgery. Cognitive deficits present barriers to care and reintegration into society [20]. Both cognitive rehabilitation and pharmacological interventions have been utilized in treating deficits in patients with PBTs. Regarding pharmacologic intervention, several classes of drugs have been explored. Stimulants such as methylphenidate were among the earliest drugs considered, but there is not a strong evidence base to support its use in this setting. Donepezil, an acetylcholinesterase inhibitor, and memantine, an NMDA-receptor antagonist, have been reported to have benefits, however [21]. Cognitive rehabilitation interventions are discussed later in this review.

### 4.3. Fatigue

Fatigue is a commonly noted problem among patients with PBTs, and many factors can contribute. A broad differential should be considered as possible causes or exacerbating factors of fatigue. Beyond organ system considerations (endocrine, cardiac, pulmonary, psychiatric, etc.), clinical treatments, sleep impairments, and a decrease in functionality should all be considered as part of the clinical picture when evaluating for fatigue. Fatigue has also been shown to be correlated with existential distress, which is present in this population at meaningful rates (25%) [16]. Although neurostimulants such as methylphenidate, modafinil, and armodafinil are commonly used pharmacologic treatments, there is not enough evidence to support the use of pharmaceutical or non-pharmaceutical interventions for fatigue in this population [38].

### 4.4. Sleep Disturbances

Sleep disturbance is a common issue for patients with PBTs, and in turn can contribute to other symptoms and psychopathology. A study of 424 patients with PBTs found that 19% of patients reported having moderate–severe sleep disturbance. The findings were associated with younger age, poor Karnofsky Performance Status, current steroid use, and tumor progression on MRI. These patients also reported greater overall symptom burden in cognition and mood, where 72% reported fatigue, 59% drowsiness, and 56% distress. In addition, there was an increased frequency of moderate–severe anxiety and depression compared to those without sleep disturbances [19]. Hypnotic medications zolpidem and trazodone have been studied in patients with PBTs suffering from insomnia after brain tumor resection and have been shown to cause significant improvements in patients’ quality of sleep [39].

### 4.5. Personality Changes

Patients can experience changes in personality that may be related to processing the grief and anxiety around their diagnoses as well as suffering from personality changes caused by the tumor itself. Personality changes could also present in the form of fatigue, anxiety, and social isolation [17]. Changes in personality should be monitored at regular intervals. There is no current qualitative or quantitative tool to accurately measure a patient’s change in personality, but information from the patient’s caretakers can be useful in tracking changes. Patients may not be aware that their personalities are changing. Early detection could lead to a timely referral to other providers such as neuropsychologists and neuropsychiatrists, and underlying causes such as surgery, radiation, and medication should be explored [40].

### 4.6. Mood Changes

Like personality changes, changes in mood may occur after the diagnosis of a brain tumor. Difficulty with processing the emotions around accepting such a life-altering diagnosis can lead to emotional outbursts, anger, and intrusive thoughts. Other mood changes, such as mania, anxiety, depression, and suicidal ideation, are also possible and have been reported. Symptoms of depression can occur in up to 15–20% of patients in the 8 months after being diagnosed. Several factors are thought to be involved, including the patient’s personal or family history of psychiatric illness, psychosocial factors, and biochemical changes in the brain parenchyma. Typically, management for depression includes a combination of pharmacological intervention and psychotherapy; however, these interventions have not been well studied in the population of PBT patients. Cognitive-behavioral therapy does not carry associated risk but may be difficult to implement if the patient is suffering from fatigue or cognitive deficits. Although in a healthy population, pharmacotherapies such as Selective Serotonin Reuptake Inhibitors (SSRIs) are not likely to induce seizures, there is little literature to verify that this is true in this population. Interventions in mindfulness, problem-solving therapy, and exercise programs can also be considered [17].

### 4.7. Hallucinations and Psychosis

A less frequently reported complication in this population is hallucinations and psychosis. If hallucinations or psychosis are suspected to be secondary to the brain tumor, pharmacological intervention with low dose first-generation antipsychotics or second-generation antipsychotics such as olanzapine and risperidone can be used. Typically, these symptoms are not seen in isolation from other psychiatric symptoms, but a strictly sensory hallucination in any modality can occur in the context of epilepsy, which can be managed with antiepileptic medications. Iatrogenic causes such as the use of steroids, antiepileptic medications, and SSRIs should be considered [17].

## 5. Goals of Inpatient and Outpatient Therapy

The rehabilitation of patients with PBTs requires a multidisciplinary effort. To address the gamut of neurological, cognitive, and physical symptoms that occur from disease progression and treatment side effects, services in both the inpatient and outpatient settings can be utilized. A retrospective analysis of 719 patients with brain tumors in Italy showed that over 12 months after initial diagnosis, 12.8% of patients went to an inpatient rehabilitation program, 11.8% utilized outpatient rehabilitation services (motor, speech, cognitive, occupational, and/or psychological), and 3.1% received an individualized intensive outpatient rehabilitation plan including motor, speech, and occupational therapy occurring 5 days a week for 3 months [41]. While these numbers are specific to the healthcare infrastructure of the Lazio region of Italy, other studies suggest that rehabilitation services may generally be underutilized by the PBT population, with unmet needs seen in the United States, United Kingdom, Norway, and Australia [42,43,44,45]. Investigators in Queensland, Australia, conducted interviews with 19 patients diagnosed with primary brain tumors and identified general reasons why rehabilitation and community service needs went unmet. They identified some patients who had a perception that support services were not needed, while others understood their unmet needs but chose not to use services, and a third group expressed the desire to engage in services but had obstacles to accessing them [46]. It is vital for healthcare providers to address the rehabilitation needs of their patients, as rehabilitation services could lead to cognitive benefits, increased functionality, and overall improvement in the health-related quality of life. The rehabilitation type and setting should be catered to the individual needs of patients and their caregivers, and when considering the devastating prognoses some brain tumors carry, the benefits of rehabilitation should be weighed against the time spent in a healthcare facility. Studies exploring rehabilitative measures in patients with primary brain tumors are further discussed below.

### 5.1. Inpatient Rehabilitation

Huang et al. aimed to show the benefit of inpatient rehabilitation in the PBT population. They examined 10 consecutive patients admitted to inpatient rehabilitation and found overall that while there were functional outcome improvements upon discharge, significant quality of life improvements only appeared at 1 and 3 months after discharge [47]. In a retrospective cohort study examining the outcomes and survival benefit of inpatient rehabilitation for 100 patients with newly diagnosed glioblastoma after surgical resection, it was found that rehabilitation improved measures of mobility, self-care, communication, and sphincter control, but the mean survival time was not significantly different compared to 312 similar patients who did not go to rehabilitation [48].

Four studies examined inpatient rehabilitation functional outcomes in PBT patients compared with stroke patients [49,50,51,52]. Of these, two were retrospective studies [49,52] and two were prospective [50,51]. Three of the studies matched stroke and tumor patients with a focus on the side of lesion and location [50,51,52] while the fourth compared 168 PBT patients with 1660 first time hemorrhagic or ischemic stroke patients [49]. All studies looked at a PBT population consisting of both benign and malignant tumors and found that the PBT population had functional gains comparable to stroke patients at discharge. Bartolo et al. found that meningioma patients achieved better mobility and independence in activities of daily living compared to patients in glioblastoma or stroke groups [51]. Yu et al. incorporated a follow-up telephone survey with caregivers that occurred 1 year or later after patients had been discharged from inpatient rehabilitation and found that most patients not only had improved or maintained function (89.5% motor, 84.2% cognition, and 84.2% ADLs) but also that caregivers were satisfied with the intensity of therapy (77%) and would recommend inpatient rehabilitation to people in similar situations (69.2%) [52].

Fu et al. compared 21 high-grade glioma patients with 21 demographically and lesion matched low-grade astrocytoma patients and found that the high-grade astrocytoma patients had longer lengths of stay and greater functional gains in inpatient rehabilitation [53].

Reilly et al. compared functional outcomes of 25 patients with initial diagnosis glioblastoma multiforme with 25 demographic- and lesion- matched recurrent glioblastoma multiforme patients and found that there was no statistical difference in functional gains at discharge between the two groups [54]. The full table can be seen in Table 2.

### 5.2. Outpatient Rehabilitation

Outpatient therapy is an avenue for rehabilitation that is available to PBT patients, but the results of its utilization have been relatively less studied compared to inpatient rehabilitation [55]. Shahpar et al. studied the benefits of an interdisciplinary outpatient rehabilitation program in a population of patients with malignant brain tumors [56]. In this prospective longitudinal study, the researchers reported that of the 49 enrolled patients, 46 completed the outpatient program with an average length of stay of 76.9 days. The therapists noted significant improvement in the total Day Rehabilitation Outcome Scale (Day-ROS) along with the specific subcategories of communication, mobility, and activities of daily living from admission to discharge. The Disability Rating Scale (DRS) was measured at admission and discharge by therapists and at 1 month and 3 months post-discharge by caregivers. No improvement was found over time. Another study conducted by Galea et al. compared 106 survivors of glioma (grades I-IV) between two groups, one receiving multidisciplinary outpatient therapy and another control that was placed on a waitlist [57]. The 53 patients receiving treatment were administered a 6–8-week individualized regimen, with sessions occurring 2–3 times per week. The primary outcome measurement was the Functional Independence Measure (FIM) score, and it was measured at baseline and 3 and 6 months after completion of the program. At 3 months, the rehabilitation group had significant improvements under several FIM subscales including motor, communication, and psychosocial. At 6 months, the rehabilitation group had maintained benefits for only the FIM elements of sphincter, communication, and cognition. Hansen et al. examined the effectiveness of a supervised physical and occupational based therapy in a population of glioma patients (grades II–IV) actively undergoing anticancer treatment [58]. Thirty-two patients were randomized into the intervention group consisting of a 6-week individually tailored physical and occupational regimen, and 32 control patients were referred to usual local rehabilitation according to their screening at hospital discharge. The primary outcome was quality of life as measured by self-ratings from the global health status (GHS) and specific domains of the European Organization for Research and Treatment of Cancer Questionnaire at baseline and 6-week follow-up. There was no significant difference across the groups at follow-up.

### 5.3. Cognitive Rehabilitation

Cognitive deficits are commonly observed in patients with brain tumors and are often found in the areas of attention, memory, and executive functioning. Due to the large amount of literature available on this topic, a brief overview of the randomized control trials specifically examining a population of primary brain tumors were selected for discussion. Studies that examined patients with brain metastases were excluded.

A randomized controlled trial looked at a population of 140 adult patients with low-grade and anaplastic gliomas with cognitive deficits who were considered to be in remission. Patients randomized into the treatment arm received 6 weekly two-hour sessions of individualized cognitive training administered by a neuropsychologist along with weekly computer-based homework assignments and one booster session three months after completion. Compared to the control arm, these patients showed moderate gains in combined attention tests and verbal memory at 6 months. Also, a higher percentage of patients no longer met the criteria to be considered as having a cognitive deficit at 6 months in the treatment arm [59]. Richard et al. compared the benefits of cognitive strategy training in a population of 25 patients with mixed primary brain tumors, both benign and malignant, and who were at least 3 months removed from radiation or surgery, if applicable. The participants were split into three groups, one receiving cognitive strategies (*n* = 11), another intervention group receiving general education and activities (*n* = 8), and a third wait-list control group (*n* = 6). Assessments were made at baseline, immediately after completing training, and 4 months after completing training. The results showed that while both intervention groups had less cognitive concerns than the control group after completing training and on follow-up, the group receiving cognitive strategy training had higher levels of functional goal attainment than the general intervention at both time points [60].

In a study by Locke et al., 19 pairs of PBT patients and their caregivers were randomized into the intervention group (*n* = 12), consisting of 6 cognitive rehabilitation sessions and 6 problem-solving sessions that were administered concurrently with radiation treatment over a 2-week period or the control group receiving only standard medical care (*n* = 7). Thirteen patients completed the study, 8 in the intervention group and 5 in the control group. The primary outcomes were improvements in quality of life and cognitive functioning as measured by surveys at baseline, post intervention, and 3-month follow-up. Patients were assessed with Functional Assessment of Cancer Therapy-Brain version (FACT-BR) for quality of life and the Mayo-Portland Adaptability Inventory-4 (MPAI-4) for functional status. The caregivers filled out a survey with MPAI-4 at these same time intervals. They found no statistical difference across groups in the patient or caregiver surveys [61]. One study looked at early cognitive rehabilitation of patients with mixed primary brain tumors who were admitted to inpatient rehabilitation. The study examined an intervention group (*n* = 30), consisting of patients who received 16 one-hour sessions of cognitive training over 4 weeks while the control group (*n* = 32) received the usual care. Cognitive assessment was done at baseline and 4 weeks later and revealed that the intervention group had significant improvement in cognitive performance over the control group [62].

### 5.4. Complementary Therapies

Complementary and Alternative Medicine (CAM) describes the use of therapies and practices with origins outside of Western Medicine. A similar approach is Integrative Medicine which combines complementary and conventional medicine in a cohesive way, often offered at the same treatment facility [63].

A 2007 NHIS study found 65% of participants who had been diagnosed with cancer used CAM, while only 53% of other participants did. In the cancer population, patients are interested in these approaches for increasing overall wellness, building immunity, and managing pain and other side effects. While patients with cancer are more likely to use CAM, the reported prevalence varies between studies due to types of cancer, age, gender, and socioeconomic status. The same NHIS study found that 15% of respondents using herbs told their providers, and only 23% told of their general use of CAM. It is important to share this information with health care providers to ensure the safety of their use and be aware of any potential interactions [64]. Armstrong et al. studied the use of CAM in patients with primary brain tumors. Of the 101 study participants, 34% reported using some form of complementary medicine, with 41% of those using multiple modalities. Of these, 74% of the patients reported not disclosing their CAM use to their physicians. The most common uses included prayer, vitamins, herbal supplements, shark cartilage, Essaic (herb blend), green tea, and faith healing. The study also found that the grade of tumor malignancy was not correlated with CAM use. Surprisingly, this study found that in self-reported quality of life, there was no significant difference between those who used CAM and those who did not [65]. Another study looked at yoga for patients with high-grade gliomas undergoing radiotherapy and their caregivers. Of the 5 dyads that consented and completed all 12 sessions, all patients and caregivers reported benefit from their participation. There were clinically meaningful reductions in patient’s cancer symptoms, as well as clinically significant decreases in patient sleep disturbances and enhancing patient and caregiver mental Quality of Life (QOL) [66].

The diagnosis of cancer can cause patients to question the meaning of life. Studies have investigated how spirituality and emotional well-being contribute to patients’ coping with their new reality. Visser et al. performed a literature review, concluding that while 31 of 36 studies demonstrated positive associations with spirituality and higher well-being, there were methodical overlap issues making it difficult to separate spirituality from emotional health [67]. Ownsworth and Nash discussed existential well-being in patients with primary brain tumors. For some, the diagnosis strengthens their spirituality, but for others, it can change their beliefs and cause them to search for meaning elsewhere [68]. Piderman et al. created a spiritual legacy intervention called “Hear my Voice” which involved chaplain-led spiritual interviews with 19 patients with PBTs. In this small sample, brain cancer was found to enrich their relationships with God or the spiritual, others, and the self. Results found “continued vitality, growth and generativity of these participants” [69]. Spirituality can be used as an additional source of support for this population.

## 6. Education

### 6.1. Communication

Patients that are receiving an initial diagnosis of a primary brain tumor are likely to experience many emotions. Combined with the possible effects of the tumor including cognitive deficits and dysphasia, the sharing of information can be complicated. Also, the shock associated with initially hearing of a PBT diagnosis could make it difficult to process prognosis and treatment options. Rehabilitation has been shown to have a role in both restorative and palliative care, and it should be discussed with PBT patients when having goals-of-care conversations. Because maintaining functionality is often important to patients, the side effects, medical complications, and recuperation from potentially invasive treatments are appropriate topics for health care providers to address. Below, we discuss literature that focuses on understanding the elements of effective communication in this population.

Halkett et al. identified four major themes in the information and support needs of patients diagnosed with high-grade gliomas including feelings of uncertainty around prognosis and quality of life, the need for individualized information, dependence on care givers due to cognitive deficits and functional losses, and communication with health professionals because of prognosis and communication difficulties [70]. Communication should be face-to-face and should include the patient and other persons involved in the patient’s care such as family members and caregivers. When sharing information regarding prognosis, treatment options, hospice, and palliative care, patient preferences regarding how much they want to know should be taken into consideration [71]. It has been considered that anxiety centered around the diagnosis of PBT is influenced by the patient’s perception of the tumor. Diaz et al. conducted a study examining the role of communication in anxiety reduction in patients with high-grade gliomas and found that patients who received more information about their illness and were thoroughly able to understand that information had lower levels of anxiety than counterparts who wanted to know less (only the critical or important aspects as opposed to all possible information) [72]. Also, because of the natural progression of many PBTs, it is important to establish decision-making capacity and ensure that a plan is in place for a health care proxy if the time comes that the patient is unable to make decisions for themselves.

### 6.2. Family Training

Boele et al. studied the relationship between mastery of caregiving by informal caregivers and the health-related quality of life (HRQOL) of patients with high-grade gliomas. They noted that in some circumstances, a patient’s partner adapted to the role of the informal caregiver and explored whether an intervention of psychoeducation and cognitive behavioral therapy for the caregiver would lead to benefits for both themselves and the patients. The study showed a relationship between patients’ HRQOL and neurological functioning and the informal caregivers’ HRQOL and feelings of mastery in skills required to provide care. In the intervention group, the informal caregivers were given one-hour sessions at baseline and every 2 months for 8 months. This group showed maintained levels of HRQOL and feelings of improved level of mastery in providing care [73]. Another preliminary study published in 2017 suggests that informal caregiver’s level of mastery could influence survival in the population of patients with glioblastoma multiforme [74].

## 7. Screening

For baseline assessment, neurocognitive screening can be done. Several options are available for the method of screening. In patients with low-grade glioma, abnormal baseline Mini-Mental State Examination (MMSE) was a predictor of poorer survival [75]. The Montreal Cognitive Assessment (MoCA) is a popular screening tool and has been shown to have a higher sensitivity for identifying cognitive deficits than the MMSE. In the population of patients with brain tumors, its applicability has been questioned because it is a measure of global cognitive functioning and does not screen for specific cognitive deficits that may be present due to the qualities and location of a tumor. In a study examining a mixed population of patients with various primary brain tumors, including both benign and malignant types, one study found that the MoCA had poor sensitivity and negative predictive value in screening for specific cognitive deficits as compared to a 1–1.5-h neuropsychological assessment. These findings suggest that even if a patient scores a normal score of 26 or greater on the MoCA, a cognitive assessment should still be considered to evaluate for specific cognitive deficits [76]. Testing for specific cognitive domains can be done with Trail Making Test Part A, Trail Making Test Part B, Digit Span, Letter-Number Sequencing, Hopkins Verbal Learning Test-Revised, Boston Naming Test, Wechsler Adult Intelligence Scale 4th edition, and Controlled Oral Word Association Test [28]. In order to screen for mood changes, healthcare providers can screen with The Hospital Anxiety and Depression Scale and the Patient Health Questionnaire-9 [17]. In addition to healthcare providers, neurocognitive and behavioral changes can be observed by family and caretakers.

## 8. Survivorship Care

Survivorship care is defined as the spectrum of care offered to patients that are living with cancer. It is focused on patient-centered approaches that fulfill the broad needs of care in these populations. The question as to how to best deliver this care has been considered by several entities. The Institute of Medicine (IOM) published a book entitled From Cancer Patient to Cancer Survivor, Lost in Transition, in which they outlined services and health care providers that could meet the needs of this population [23]. The American Society of Clinical Oncology (ASCO) explored different models of delivering care [24]. Rosenzweig et al. explored the interprofessional management of cancer survivorship, detailing the members and roles of providers in their delivery of survivorship care, with a consideration of the potential expansion in the utilization of nurse practitioners and physician assistants [25]. Recently, Leeper and Milbury proposed a model of survivorship care specific to the neuro-oncology population [22]. They outlined a patient-centered approach that focuses on addressing the needs and symptoms as reported by outcome assessments in trials, as defined by the FDA. Their model looks to focus on stakeholders in the patient’s care, such as healthcare providers, family, and patient advocacy groups. Within this system, they looked at the way these characters interacted with one another and the effect that had on patient outcomes, specifically focusing on care coordination, provider-to-patient communication, and patient-to-provider communication. They outlined desired impacts of survivorship care to include management of comorbid conditions, management of psychosocial conditions, management of long-term and late effects of disease and its treatment, adherence to surveillance, optimizing the use of health care resources, and engagement with health resources available at local, state, and federal levels. They propose that this could lead to benefits in health-related quality of life, mood, fatigue, functional status, cognitive status, and seizure, headache, and pain control.

## 9. Hospice

Current guidelines for referral to hospice care involve patients with life expectancy less than six months, those have poor or worsening performance status, those who are not candidates for surgery or chemotherapy, those who have deteriorating neurologic functions despite therapy, or those who have tumor recurrence [4]. The utilization of these services has shown to have numerous benefits, both for patients and for caregivers, although it has been documented that they are underused. In two retrospective cohort studies, late hospice referrals (within 7 days or sooner before death) were noted to occur 22–24% of the time in patients with primary malignant brain tumors [77,78]. Patients with later referrals were more debilitated and not able to achieve maximal benefit from hospice services. Risk factors that were associated with late referrals included male gender, lower socioeconomic status, and lack of a healthcare proxy [77]. Medicare expenditures were examined in older adults, and hospice utilization led to a mean reduction in approximately $12,000 in healthcare costs [78].

## 10. Discussion

Primary brain tumors can be devastating diseases that pose unique challenges for patients and their care providers. There are several elements to be considered when offering care, keeping the overall quality of life in mind. Providers should address common concerns in this population including complications from chemotherapy and radiotherapy, neurologic symptoms, cognitive deficits, and psychological changes. Many of these presentations can be relieved with the use of medications. When evaluating the patient’s rehabilitation goals, the pros and cons of inpatient and outpatient settings should be considered, especially for patients with poorer prognoses. Studies of inpatient rehabilitation in this population demonstrated functional improvements at the end of the intervention. Cognitive rehabilitation was also shown to improve patients’ cognitive deficits. Complementary and alternative methods have also been utilized by this population to increase overall wellness, though a majority of patients do not disclose this information to their providers. The diagnosis and subsequent treatment and rehabilitation of primary brain tumor can be a great deal of information for patients and caregivers to process. Therefore, special attention should be given to communication challenges, family training, and potential referrals to hospice care. The journey of patients with primary brain tumors is a continuum from diagnosis to treatment to rehabilitation to hospice, with many stakeholders involved at each point. Physiatrists have a role in the spectrum of survivorship care and should be viewed as members of interdisciplinary teams. Together, the team can work towards achieving the patient’s goals and enriching their quality of life.

## Figures and Tables

**Table 1:** Demographics of common primary brain tumors in the United States from 2012–2016.

	5-Year Total	% of Tumors	Median Age	M:F Annual
Meningioma	152,756	37.6%	66.0	1:2.72
Sellar Region Tumors	71,084	17.5%	51.0	1:1.20
Glioblastoma	59,164	14.6%	65.0	1.37:1
Nerve Sheath Tumors	35,017	8.6%	56.0	1:1.09
Unclassified Tumors	20,556	5.1%	63.0	1:1.20
Lymphoma	7680	1.9%	66.0	1.02:1
Diffuse Astrocytoma	7500	1.8%	47.0	1.22:1
Anaplastic Astrocytoma	7015	1.7%	53.0	1.23:1
Ependymal Tumors	6877	1.7%	45.0	1.32:1
Pilocytic Astrocytoma	5166	1.3%	12.0	1.08:1
Oligodendroglioma	3668	0.9%	43.0	1.21:1
Embryonal Tumors	3493	0.9%	8.0	1.41:1
Germ Cell Tumors	1543	0.4%	16.0	2.18:1
Choroid Plexus Tumors	817	0.2%	36.0	1.01:1
Pineal Region Tumors	796	0.2%	34.5	1:1.34

Based on CBTRUS Statistical Report: U.S. Cancer Statistics—NPCR and SEER 2012–2016. These values make up 94.4% of tumors from the data collected from 2012–2016.

**Table 2:** Comparison of inpatient rehabilitation for patients with primary brain tumors.

Author	Design	Outcome Measures	Outcome
Huang et al. 2001 [47]	Prospective study with longitudinal data examining 10 consecutive patients with primary brain tumors admitted to inpatient rehabilitation.	Outcome measures included Functional Independence Measurement (FIM), Disability Rating Scale (DRS), Karnofsky Performance Status Scale (KPS), and Functional Assessment of Cancer Therapy—Brain (FACT-BR) at admission, discharge, and 1 and 3-month follow-ups.	Functional outcome improvement was noted by all functional measures (FIM: F = 46.84, *p* < 0.05; DRS: F = 19.25 *p* < 0.05; KPS: F = 10.11, *p* < 0.05) Quality of life improvement as measured by FACT-BR only had significant improvement between admission 1-month and admission 3-month follow-up scores (F = 6.75 *p* < 0.05)
Greenberg et al. 2006 [49]	Retrospective study examining 168 primary brain tumor patients (128 intracranial meningiomas and 40 cerebral gliomas) compared with 1660 first time hemorrhagic or ischemic stroke patients.	Onset-to-admission interval, functional status at admission and discharge, length of stay, functional gain as measured by Functional Independence Measurement (FIM) between admission and discharge.	On average, meningioma patients were admitted 13 days after excision, glioma patients 34 days after surgery, and 21.6 days for stroke patients. Baseline and discharge FIM ratings were 80.07–90.3 with a functional gain of 17.9 for meningioma patients, 68.2–80.7 with a functional gain of 17.2 for glioma patients, and 70.4–87.8 with a functional gain of 21.8 for stroke patients. Length of stay in days was 24, 23, and 75.4 for meningioma, glioma, and stroke patients respectively
Geler-Kulcu et al. 2009 [50]	Prospective comparative study examining 21 patients with intracranial tumors (6 meningiomas and 15 glial tumors) compared to 21 patients with ischemic or hemorrhagic strokes who had matching lesions to the brain tumor patients.	Motor Assessment Scale (MAS), Postural Assessment Scale for Stroke (PASS), Berg Balance Scale (BBS), Functional Independence Measurement (FIM) measured at admission and discharge.	Both the brain tumor and stroke group made improvements in all measures (MAS, PASS, BBS, and FIM) from admission to discharge but there was no significant difference in the extent of improvements made between the two groups.
Fu et al. 2010 [53]	Retrospective comparative study examining 21 high-grade glioma patients admitted to compared to 21 low grade astrocytoma patients who were demographic- and lesion-matched.	Functional gain from admission to discharge as measured by Functional Independence Measurement (FIM) and length of stay in inpatient rehabilitation.	High-grade astrocytoma patients had longer lengths of stay (13 days compared to 9 days *p* = 0.0384) and greater overall FIM gains (21.7 compared to 13 *p* = 0.0181) than low-grade astrocytoma patients.
Bartolo et al. 2012 [51]	Prospective case-controlled study examining 75 patients who had undergone neurosurgery for primary brain tumors (32 meningiomas and 43 glioblastomas) compared to 75 acute hemorrhagic or ischemic stroke patients matched both by demographic and side of lesion, with focus on lobes affected by vascular distribution with respect to lobes affected in the brain tumor group.	Functional Independence Measure (FIM) score, Sitting Balance score, Standing Balance score, Hauser Index, Massachusetts General Hospital Functional Ambulation Classification (MGHFAC) of patients at time of admission and time of discharge 4 weeks later.	Both groups had significant improvements in all outcome measures as measured from admission to discharge (*p* = 0.000 for all measures) No significant difference was present as a measure of improvement between the brain tumor and stroke groups as a whole, but subgroup analysis showed the meningioma patients achieved better results in independence in activities of daily living (*p* = 0.02) and mobility (*p* = 0.04) compared with patients in the glioblastoma or stroke groups.
Roberts et al. 2014 [48]	Retrospective cohort study examining 100 patients with newly diagnosed glioblastoma multiforme status-post surgical resection admitted to inpatient rehabilitation compared to a similar group of 312 newly diagnosed glioblastoma multiforme patients status-post surgical resection that did not get admitted to inpatient rehabilitation.	Survival time and functional improvement as measured by Functional Independence Measure (FIM) instrument within 3 days of admission and 3 days of discharge.	After adjusting for confounding variables, there was no significant difference in survival times between the patients who went to inpatient rehabilitation and those who did not. 93.7% of patients who underwent inpatient rehabilitation had improved functional status and an average total FIM score improvement of 19.7 (*p* < 0.0001). When examining specific FIM scales, mobility, self-care, cognition, and sphincter control had average improvements of 8.3, 7.1, 2.3, and 1.9 points respectively (*p* < 0.0001 for each scale).
Yu et al. 2019 [52]	Retrospective study examining 35 patients with brain tumors, 21 benign (11 WHO grade I, 10 WHO grade II) and 14 malignant (4 WHO grade III and 10 WHO grade IV) admitted to inpatient rehabilitation compared with 108 stroke patients admitted to inpatient rehabilitation that were matched demographically, by lesion location, and by length of stay.	Functional improvements in motor, cognition, and activities of daily living from admission to discharge as measured by Fugl-Meyer Assessment (FMA), Berg Balance Scale (BBS), Korean version of the modified Barthel Index (K-MBI) score, Korean Mini-Mental State Examination (K-MMSE) score, and intelligence quotient (IQ) score. In the brain tumor group, Eastern Cooperative Oncology Group (ECOG) scale was measured to confirm functional level. Telephone survey after 1 year evaluating caregivers on their opinion of rehabilitation.	Within the brain tumor and stroke groups, gains were seen in FMA score, BBS score, K-MBI score, K-MMSE score, and IQ score (*p* < 0.001 for each) and there were no significant differences between groups for these outcomes. Subgroup analysis of benign and malignant tumor groups found improvement in ECOG scale (*p* < 0.001 for benign, *p*< 0.011 for malignant) with no significant difference between groups in any outcome measure. Caregiver survey occurring on average more than 2 years after discharge revealed that patients had improved or maintained function (motor 89.5%, cognition 84.2%, and ADL 84.2%) and that 77% were satisfied with the intensity of therapy and 69.2% would recommend inpatient rehabilitation to patients in similar positions.
Reilly et al. 2020 [54]	Retrospective case matched study comparing 25 patients with an initial diagnosis of glioblastoma multiforme with 25 demographic- and lesion-matched patients who had a diagnosis of recurrent glioblastoma multiforme.	Functional outcomes measured by Functional Independence Measure (FIM) at admission and discharge.	There was no statistically significant difference between the groups for FIM scores at admission or discharge, or overall FIM gains or efficiencies. The study also found no significant differences in rates of complications while in inpatient rehabilitation.
